# Exploiting Dual-Attention Networks for Explainable Recommendation in Heterogeneous Information Networks

**DOI:** 10.3390/e24121718

**Published:** 2022-11-24

**Authors:** Xianglin Zuo, Tianhao Jia, Xin He, Bo Yang, Ying Wang

**Affiliations:** 1 College of Computer Science and Technology, Jilin University, Quanjin Street, Changchun 130012, China; 2 Key Laboratory of Symbolic Computation and Knowledge Engineering, Jilin University, Quanjin Street, Changchun 130012, China; 3 School of Artificial Intelligence (SAI), Jilin University, Quanjin Street, Changchun 130012, China

**Keywords:** heterogeneous information networks, dual attention mechanism, rating prediction, meta-path

## Abstract

The aim of explainable recommendation is not only to provide recommended items to users, but also to make users aware of why these items are recommended. Traditional recommendation methods infer user preferences for items using user–item rating information. However, the expressive power of latent representations of users and items is relatively limited due to the sparseness of the user–item rating matrix. Heterogeneous information networks (HIN) provide contextual information for improving recommendation performance and interpreting the interactions between users and items. However, due to the heterogeneity and complexity of context information in HIN, it is still a challenge to integrate this contextual information into explainable recommendation systems effectively. In this paper, we propose a novel framework—the dual-attention networks for explainable recommendation (DANER) in HINs. We first used multiple meta-paths to capture high-order semantic relations between users and items in HIN for generating similarity matrices, and then utilized matrix decomposition on similarity matrices to obtain low-dimensional sparse representations of users and items. Secondly, we introduced two-level attention networks, namely a local attention network and a global attention network, to integrate the representations of users and items from different meta-paths for obtaining high-quality representations. Finally, we use a standard multi-layer perceptron to model the interactions between users and items, which predict users’ ratings of items. Furthermore, the dual-attention mechanism also contributes to identifying critical meta-paths to generate relevant explanations for users. Comprehensive experiments on two real-world datasets demonstrate the effectiveness of DANER on recommendation performance as compared with the state-of-the-art methods. A case study illustrates the interpretability of DANER.

## 1. Introduction

Recommendation systems have been widely used in various online services, such as search engines, e-commerce, online news, and social media sites, and have become one of the most powerful ways to solve the problem of information overload [1,2]. However, a large number of recommendation methods are still black-boxes that do not provide explanations for users. In recent years, explainable recommendation has attracted increasing attention in the academic and industrial communities. Explainable recommendation systems not only unveil the recommendation process, but also help to improve the effectiveness, persuasiveness and satisfaction of the recommendations.

Traditional recommendation methods, e.g., matrix factorization, mainly infer the preferences of users for items by using implicit or explicit user–item interaction data [3]. The key to generating accurate recommendation results is to obtain the representations of users and items with rich expressive power, while these traditional methods suffer from the sparseness of interaction data [4,5]. A common idea which can solve the problem of data sparseness is to introduce some auxiliary information into the recommendation system. Auxiliary information can make up for the sparseness or lack of interaction data, enrich preferences of users and features of items and enhance the performance of the recommendation system effectively [6]. What is more, traditional recommendation methods only provide some simple explanations, such as *“Customers Who Bought This Item Also Bought…”*, with which users are not satisfied in general.

Fortunately, various kinds of auxiliary information have become increasingly available in online services. This auxiliary information can be easily organized into heterogeneous information networks. Heterogeneous information networks contain rich attribute information and semantic associations, so can provide potential relations between users and items for recommender systems [7]. By connecting different kinds of relations in heterogeneous information networks, latent higher-order interaction information between users and items can be discovered. The emerging success of mining heterogeneous information networks may shed some light on solving these issues of data sparseness and simple explanation in the recommendation system. Many existing models [8] regard reviews, an item’s aspects and meta-paths as contextual information about the user–item interaction and leverage them to improve the recommendation performances and generate recommendation explanations. Explainable recommendation has also attracted remarkable attention in recent years [9,10].

Although the above methods have achieved a better performance, there are two challenges in applying heterogeneous information networks to recommender systems: (1) how to extract effective information that can be used in recommendation systems from heterogeneous information networks; (2) how to effectively integrate high-order interaction information for better recommendation results and explanations. In order to solve the first challenge, we intend to design multiple different types of meta-paths for heterogeneous information network architectures to produce corresponding similarity matrices [11]. As for auxiliary information, it can tackle the issue of the sparseness of the original user–item interaction matrix. Then, the latent representations of users and items are obtained through matrix decomposition methods [12]. Aiming at the second issue, we present a dual-attention network to distinguish the contribution of each representation from different meta-paths to the final representations of users and items. Then, the dual-attention networks will aggregate the representations from multiple meta-paths through the attention coefficients to generate the final representations of users and items.

In this paper, we propose a framework of explainable recommendation by exploiting dual-attention networks in heterogeneous information networks (DANER), to capture the latent representations of user preferences and item features, and to learn the joint representation of user–item interactions using the dual-attention networks for the recommendation predictions and explanations. The contributions of this paper are summarized as follows:

- In order to alleviate the problem of data sparseness, we extracted multiple kinds of meta-paths between users and items from the heterogeneous information networks and generated multiple similarity matrices, which were used as complements of the rating matrix. Then, we decomposed the similarity matrices by matrix decomposition to obtain the multiple representations of users and items corresponding to different meta-paths;
- We propose a novel dual-attention network for explainable recommendation in heterogeneous information networks (DANER). It leverages a local attention layer to learn the representations of users and items, and a global attention layer to learn the joint representations of user–item interactions, both of which integrate multiple groups of different meta-path information. An attention mechanism helps to improve the explainability of the recommendation;
- We demonstrate better rating prediction accuracy than the state-of-the-art methods by performing comprehensive experiments on two benchmark datasets. In addition, by providing a critical meta-path based on attention coefficient, we show a case study on the explainability of DANER.

The rest of this paper is organized as follows: Section 2 highlights the related work of typical recommendation methods; HIN in recommendation and attention mechanisms, respectively. Section 3 introduces the definition and problem formulation. Section 4 presents the details of our proposed DANER model. Section 5 shows the experimental results. Finally, Section 6 concludes this paper.

## 2. Related Works

### 2.1. Recommendation

At present, there are two main streams of recommendation system models, one is based on collaborative filtering and the other is based on content [13]. The core idea of collaborative filtering model is to recommend items for users according to the preferences of a group of users with similar interests and common experiences [14]. Collaborative filtering recommendation algorithms can be divided into two categories: user-based collaborative filtering and item-based collaborative filtering. Matrix factorization (MF) [15] is one of the most widely used methods, which factorizes a high-dimensional original matrix into two low-dimensional matrices and uses the two new matrices to calculate the prediction rating. There are many extended works based on matrix factorization, including Probability Matrix Factorization [16], BPRMF [17]. The Probabilistic Matrix Factorization (PMF) model scales linearly with the number of observations and performs well on the large, sparse, and very imbalanced Netflix datasets, including an adaptive prior on model parameters, which shows how the model’s capacity can be controlled automatically. BPRMF presents a generic optimization criterion BPR-Opt for personalized ranking that is the maximum posterior estimator derived from a Bayesian analysis of the problem, which also provides a generic learning algorithm for optimizing models with respect to BPR-Opt. Factorization Machines (FMs) [18] introduce a new model class that combines the advantages of Support Vector Machines with factorization models. It is a general predictor working with any real valued feature vector, which model all interactions between variables using factorized parameters. SVD++ [19] introduces a new neighborhood model with an improved prediction accuracy, which models neighborhood relations by minimizing a global cost function.The new neighborhood model adds a global average rating, item rating bias, user rating bias, and interest preference between user and item. Content-based recommendation mainly generates recommendations based on item characteristics and user profiles. The factorization machine is the typical representative among them, mainly to solve the problem of feature combination under the condition of sparse data.

### 2.2. HIN in Recommendation

Heterogeneous information networks have been proposed as a general representation of a graph or network in many real world scenarios [20,21]. Because of their remarkable ability to represent heterogeneous data, they have been used in a large number of tasks of data mining, such as clustering, classification, link prediction, representation learning and similarity measurement [22,23]. Recently, some recommendation systems have used heterogeneous information networks as auxiliary information, which has achieved great success [24]. In heterogeneous information networks, a sequence composed of different types of nodes is defined as a meta-path, which is capable of extracting the relation information [25,26]. HeteMF [27] is a matrix factorization based model which takes advantage of both rating data and the related information network, and uses meta-path-based similarity as a regularization term to enhance the effect of the recommendation model. HeteRec [28] proposes to combine heterogeneous relationship information for each user differently and introduces meta-path-based latent features to represent the connectivity between users and items along different types of paths. SemRec [29] is the first to propose weighted HIN and weighted meta-path concepts to subtly depict the path semantics through distinguishing different link attribute values. SemRec not only flexibly integrates heterogeneous information but also obtains prioritized and personalized weights representing user preferences on paths. FMG [30] first introduces the concept of the meta-graph to HIN-based recommendation and then solves the information fusion problem with a “matrix factorization (MF) + factorization machine (FM)” approach.

### 2.3. Attention Mechanism

When human beings observe a scene, they always focus on some objects in the scene according to the guidance of the information they want to obtain. Inspired by this, researchers introduced an attention mechanism into machine learning and achieved remarkable results [31]. The core purpose of the attention mechanism is to select the most critical information for the current task from a large amount of information [32,33]. Nowadays, the attention mechanism has been widely used in various fields of deep learning, such as image processing, speech recognition, natural language processing, and recommendation [34,35,36]. ACF [37] introduced a novel attention mechanism in CF to address the challenging item-level and component-level implicit feedback in the recommendation. The model consists of two attention modules—the component-level attention module to select informative components of multimedia items and the item-level attention module to score item preferences. Graph attention networks (GATs) [38] present a novel neural network architecture that operates on graph-structured data, leveraging masked self-attentional layers to address the shortcomings of prior methods based on graph convolutions or their approximations. KGAT [39] proposes a new method named Knowledge Graph Attention Network that explicitly models the high-order connectivities in KG in an end-to-end fashion. It recursively propagates the embeddings from the node’s neighbours to refine the node’s embedding, and employs an attention mechanism to discriminate the importance of the neighbours. HGAT [40] first proposed a novel heterogeneous graph neural network based on hierarchical attention, including node-level and semantic-level attentions, in which the contribution of node and meta-path can be fully considered.

## 3. Problem Statement

### 3.1. Definitions

There are several definitions of heterogeneous information networks. In this paper, we introduce the definitions of HIN, the network schema and the meta-path. Next, we will illustrate these three definitions in detail.

**Definition** **1** (Heterogeneous Information Networks). *HIN is defined as a graph G=(V,E) with an object type mapping function φ:V→A and a relation type mapping function ψ:E→R, where each object v∈V belongs to a specific object type φ(v)∈A, and each relation e∈E corresponds to a specific relation type ψ(e)∈R, where the number of object types A>1 or relation types R>1.*

**Figure 1:**
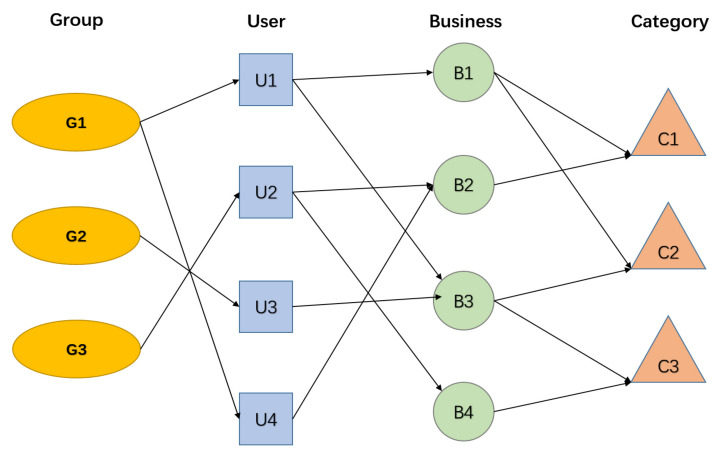
A toy example of HIN.

An example of the heterogeneous information networks is shown in Figure 1. There are four object types and three relation types in the heterogeneous information networks. The four object types are group, user, business and category. The relation between group and user indicates that a user belongs to a group. The relation between user and business indicates that a user prefers a business. The relation between business and category indicates that a business belongs to a category.

**Definition** **2** (Network Schema). *The network schema is a meta template of heterogeneous networks G=(V,E) including object mapping function φ:V→A and relation type mapping function ψ:E→R. Network schema is defined as a directed graph composed of object types A and relation types R, denoted as $T_{G}=\left(A,R\right)$.*

**Figure 2:**
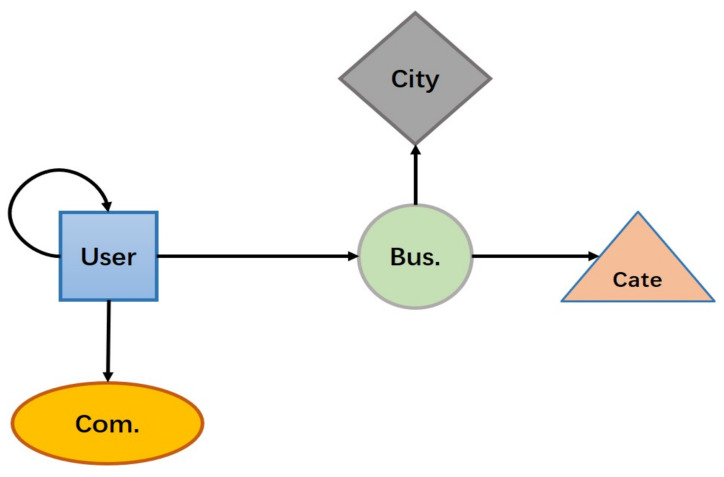
Network schema of Yelp dataset.

Figure 2 illustrates the network schema corresponding to the Yelp dataset. The Yelp dataset has five object types and five relation types. There may be more than one meta-path between two objects in an HIN. For example, user and business can be connected via U→B←U→B or U→B→Cate←B. These paths are called meta-paths, defined in Definition 3.

**Definition** **3** (Meta-path). *A meta-path is a path defined in the network schema with a starting node and a target node, such as $A_{1}\overset{r_{1}}{\to }A_{2}\overset{r_{2}}{\to }\ldots A_{n-1}\overset{r_{n-1}}{\to }A_{n}$, where $A_{i}\in A$ is the type of different object and $r_{i}\in R$ is the relation between the two objects. Apparently, the complex relation between node $A_{1}$ and node $A_{n}$ can be represented in meta-path, denoted as $R=r_{1}\circ r_{2}\ldots \circ r_{n}$, where the number of relation $R_{i}$ is the length of the meta-path.*

### 3.2. Problem Statement

For inputs to our framework, we have the user set $U=\{u_{1},u_{2},\ldots ,u_{U}\}$, the business set $B=\{b_{1},b_{2},\ldots ,b_{B}\}$, and the relation set $R=\{r_{1},r_{2},\ldots ,r_{R}\}$, where $r_{i}$ is the relation between two objects which can be user, business, category, city and so on. When the $r_{i}$ represents the relation of user and business, the weight between them indicates the rating of user on business. We design multiple meta-paths $MP_{i}$, and obtain multiple similarity matrices $M_{i}$ through the meta-paths. For the output of our framework, we provide the predicted rating ${\hat{r}}_{ui}$ of user on business and a meta-path-level explanation.

Accordingly, the two main tasks of DANER can be summarized as: (1) obtaining more expressive presentations of user preferences and item features through auxiliary information in heterogeneous information networks; (2) using the attention mechanism to aggregate these representations to get better recommendation results and providing some explanations based on the attention coefficient simultaneously.

## 4. Framework

**Figure 3:**
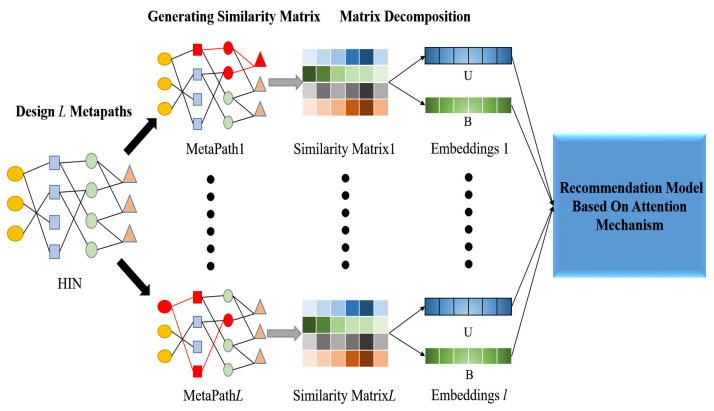
The overall structure of DANER.

In this section, we mainly introduce the use of auxiliary information in HIN and the establishment of recommendation model based on double attention mechanism. The overall structure of DANER is shown in Figure 3. DANER mainly includes three parts: the Similarity Matrices Generation, the Matrix Decomposition and the Recommendation model based on Attention Mechanism.

### 4.1. Similarity Matrix Based on Meta-Path

For a recommendation system, the starting node of the meta-path is user *u*, and the target node is item *i*. The meta-path $MP_{ui}$ represents the high-order relation between user *u* and item *i*. For example, the meta-path $u_{1}\overset{Buy}{\to }i_{1}\overset{BelongTo}{\to }Cate_{1}\overset{BelongTo}{\leftarrow }i_{2}$ in Amazon dataset indicates that user $u_{1}$ has purchased item $i_{1}$, and item $i_{1}$ and item $i_{2}$ belonging to the same category. The similarity matrix based on the meta-path is defined as $M_{MP}=M_{A_{1}A_{2}}⊗M_{A_{2}A_{3}}\ldots ⊗M_{A_{n-1}A_{n}}$, where $M_{A_{n-1}A_{n}}$ represents the relation matrix between the object type $A_{n-1}$ and $A_{n}$, ⊗ is a matrix multiplication operation between the two relation matrices.

*L* user–item similarity matrices can be obtained by *L* pre-designed meta-paths. The meta-paths of different datasets used in the experiment are shown in Table 1. For example, the meta-path used in the dataset Amazon U→B←U→B indicates that users will buy other items that have been purchased by users with the same preferences, which can be regarded as a user-based collaborative filtering. Thus, the similarity matrix corresponding to this meta-path can be obtained by the following formula $M_{UBUB}=M_{UB}⊗M_{BU}⊗M_{UB}$. Besides, Cate refers to the categories to which the item belongs, Brand refers to the brand to which the item belongs, View refers to which other items have been viewed by users who have rated the item, Com refers to the number of compliments a user receives from other users, and City refers to the city in which the restaurant is located. The calculation of the relevant meta-path is similar to the procedure mentioned above.

### 4.2. Latent Representation by Matrix Decomposition

The recommendation learning process can be regarded as a representation learning process [41]. After obtaining user–item similarity matrices corresponding to *L* meta-paths, we adopted matrix decomposition to obtain the latent representations of users and items. By using low-dimensional vector of the latent representation, we can reduce noise and alleviate the data sparseness problem of the original rating matrix [42]. Based on the theory of matrix decomposition, the similarity matrix *M* can be decomposed into two low rank matrices $I_{U}$ and $I_{B}$, where $I_{U}$ represents the latent features of users’ preferences and $I_{B}$ represents the latent features of items. Then we can use $\hat{M}=I_{U}\times I_{B}$ to generate the prediction similarity matrix $\hat{M}$. By reducing the difference between *M* and $\hat{M}$, we can obtain the latent representation matrices $I_{U}$ and $I_{B}$, which can represent the latent features of users and items better. To be specific, low-dimensional representations of users and items can be obtained by solving the following optimization problem:

(1) $$\min_{U,B}{\left(\hat{M}-M\right)}^{2}+\lambda_{1}{∥I_{U}∥}_{F}^{2}+\lambda_{2}{∥I_{B}∥}_{F}^{2},$$

where $\lambda_{1}$ and $\lambda_{2}$ are dynamic parameters, which are used to control the influence of Frobenius norm regularization to avoid overfitting. The goal of optimization is to make $I_{U}$ and $I_{B}$ restore the similarity matrix *M* as complete as possible.

For *L* similarity matrices based on meta-paths, we can obtain *L* groups of feature representations of users and items $I_{U}^{\left(1\right)},I_{B}^{\left(1\right)},I_{U}^{\left(2\right)},I_{B}^{\left(2\right)}\ldots I_{U}^{\left(L\right)},I_{B}^{\left(L\right)}$ by performing a matrix decomposition operation.

### 4.3. Recommendation Model Based on Attention Mechanism

**Figure 4:**
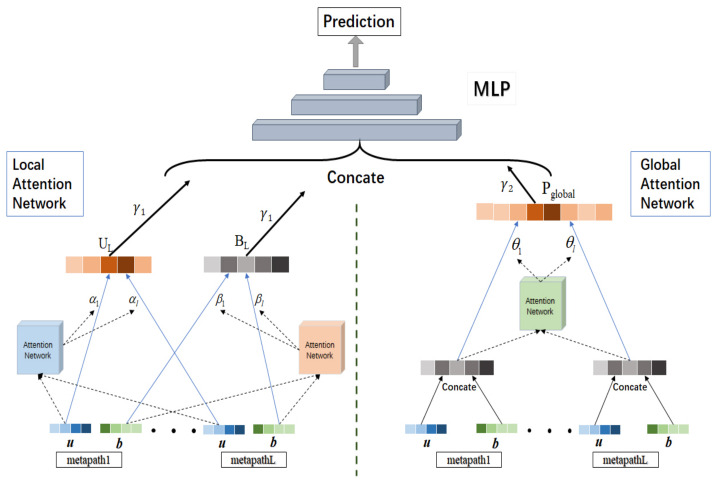
Recommendation model based on attention mechanism.

After obtaining *L* groups of representations of users and items, we also need to fuse them to obtain more expressive representations of users and items. Thus, at first, we designed a model including two attention networks to integrate these representations. The local attention network was oriented to each user (item), which was used to distinguish the importance of each user (item) representation corresponding to different meta-paths. According to the weighted combination of attention coefficients, the representations of users and items integrating *L* groups of meta-path information can be obtained respectively. The global attention network is oriented to each meta-path, which is capable of discriminating the importance of each user–item joint representation corresponding to different meta-paths. Besides, the attention coefficients can be used to select the meta-path that has the most influence on the final prediction results. By way of the global attention network, we can obtain the user–item joint representations integrating *L* groups of meta-path information. Then, the representations obtained from the two attention networks are concatenated as the input of next part. Finally, we can utilize a multi-layer perceptron to generate the prediction ratings. The specific recommendation model is shown in Figure 4, mainly including three parts, which will be introduced separately below.

#### 4.3.1. Local Attention Network

The goal of local attention network is to learn the representations of users and items, which integrate *L* groups of representations corresponding to different meta-paths. The input of local attention network is *L* groups of representations of user and item obtained by matrix decomposition. Each group of representations contains user representation $I_{U}^{\left(i\right)}$ and item representation $I_{B}^{\left(i\right)}$. For *L* groups of user representations $I_{U}^{\left(i\right)}$(i=1,…,L), we feed them into the user-oriented attention neural network to obtain the attention coefficient $\alpha_{i}$ corresponding to $I_{U}^{\left(i\right)}$:

(2) $$DNNu_{i}=Relu\left(W_{U}^{\left(n\right)}\times \cdots \times Relu\left(W_{U}^{\left(1\right)}\times I_{U}^{\left(i\right)}+B_{U}^{\left(1\right)}\right)+\cdots +B_{U}^{\left(n\right)}\right),$$

(3) $$\alpha_{i}=\frac{e^{DNNu_{i}}}{\sum_{i\in L}e^{DNNu_{i}}},\left(i=1,\ldots ,L\right),$$

where DNNu is a user-oriented attention neural network. To be specific, the input of DNNu is user representations from different meta-paths, and its output is attention scores. $W_{U}^{\left(i\right)}$ and $B_{U}^{\left(i\right)}$ are parameter matrix and bias term of the fully connected neural network of layer *i*, and we use Relu as the activation function of each layer. Then, to compute the attention coefficient $\alpha_{i}$, the Softmax function is introduced to normalize *L* output values of the neural network.

By adopting the same operation for item, the attention coefficient $\beta_{i}$ corresponding to the item $I_{B}^{\left(i\right)}$(i=1,…,L) from different meta-paths can be obtained as follows:

(4) $$DNNb_{i}=Relu\left(W_{B}^{\left(n\right)}\times \cdots \times Relu\left(W_{B}^{\left(1\right)}\times I_{B}^{\left(i\right)}+B_{B}^{\left(1\right)}\right)+\cdots +B_{B}^{\left(n\right)}\right)$$

(5) $$\beta_{i}=\frac{e^{DNNb_{i}}}{\sum_{i\in L}e^{DNNb_{i}}},\left(i=1,\ldots ,L\right).$$

Then, according to the obtained attention coefficients $\alpha_{i}$ and $\beta_{i}$, we can combine *L* groups of representations of user (item) from different meta-paths to produce $U_{L}$ ($B_{L}$). The adopted combination method is to multiply the user (item) representation with the corresponding attention coefficient $\alpha_{i}$($\beta_{i}$), and then directly concatenate the *L* groups of $\alpha_{i}\times I_{U}^{\left(i\right)}$($\beta_{i}\times I_{B}^{\left(i\right)}$):

(6) $$U_{L}=Concate\left(\alpha_{1}\times I_{U}^{\left(1\right)},\alpha_{2}\times I_{U}^{\left(2\right)}\ldots ,\alpha_{L}\times I_{U}^{\left(L\right)}\right)$$

(7) $$B_{L}=Concate\left(\beta_{1}\times I_{B}^{\left(1\right)},\beta_{2}\times I_{B}^{\left(2\right)}\ldots ,\beta_{L}\times I_{B}^{\left(L\right)}\right).$$

The local attention network layer can generate user representation $U_{L}$ and item representation $B_{L}$, which contain different meta-path information and focus on the critical meta-path information. The degree of reservation of meta-path information depends on the value of attention coefficient, the larger the attention coefficient is, the more meta-path information will be retained.

Finally, we can concatenate the user representation $U_{L}$ and item representation $B_{L}$ to obtain the local user–item joint representation $P_{local}$, which is a part of the input vector of multi-layer perceptron in the interaction model, as shown below:

(8) $$\begin{array}{cc} P_{local} & =Concate(U_{L},B_{L})\\ \; & =(\alpha_{1}\times I_{U}^{\left(1\right)}\alpha_{2}\times I_{U}^{\left(2\right)}\ldots \alpha_{L}\times I_{U}^{\left(L\right)}\\ \; & \;\;\;\;\;\beta_{1}\times I_{B}^{\left(1\right)}\beta_{2}\times I_{B}^{\left(2\right)}\ldots \beta_{L}\times I_{B}^{\left(L\right)}). \end{array}$$

#### 4.3.2. Global Attention Network

The global attention network focuses on distinguishing the contributions of user–item joint representations corresponding to different meta-paths. Firstly, we concatenate the representations $I_{U}^{\left(i\right)}$ and $I_{B}^{\left(i\right)}$ to obtain the user–item joint representation $p_{joint}^{\left(i\right)}$, where $p_{joint}^{\left(i\right)}=I_{U}^{\left(i\right)}I_{B}^{\left(i\right)}$(i=1,…,L). Then, we feed the *L* groups of $p_{joint}^{\left(i\right)}$ into the path-oriented neural network DNNp to compute the corresponding attention coefficient $\theta_{i}$:

(9) $$DNNp_{i}=Relu\left(W_{P}^{\left(n\right)}\times \cdots \times Relu\left(W_{P}^{\left(1\right)}\times p_{joint}^{\left(i\right)}+B_{P}^{\left(1\right)}\right)+\cdots +B_{P}^{\left(n\right)}\right)$$

(10) $$\theta_{i}=\frac{e^{DNNp_{i}}}{\sum_{i\in L}e^{DNNp_{i}}},\left(i=1,\ldots ,L\right),$$

where $W_{P}^{\left(i\right)}$ and $B_{P}^{\left(i\right)}$ are parameter matrix and bias terms of the fully connected neural network of layer *i*, the input of DNNp is *L* groups of user–item joint representations $p_{joint}$, the output is attention scores. Besides, Relu is used as the layer activation function in the neural network. After that, to obtain the attention coefficients θ, we introduce the Softmax function to normalize the *L* output values of the neural network.

Finally, according to the obtained attention coefficients θ, we combine the user–item joint representations $p_{joint}$ from *L* groups of meta-paths to obtain the global user–item joint representation $P_{global}$. Here, we propose to multiply the *L* groups of user–item joint representations $p_{joint}$ with the corresponding attention coefficients θ. Then, we concatenate *L* groups of $\theta_{i}\times p_{joint}^{\left(i\right)}$ to obtain $P_{global}$ directly:

(11) $$P_{global}=Concate\left(\theta_{1}\times p_{joint}^{\left(1\right)},\theta_{2}\times p_{joint}^{\left(2\right)}\ldots ,\theta_{L}\times p_{joint}^{\left(L\right)}\right).$$

Based on the attention coefficients of global attention network, we can explain the recommendation results more sufficiently, that is, the meta-path with large attention coefficient contributes more to the recommendation result.

#### 4.3.3. Interaction Model

After obtaining the local user–item joint representation $P_{local}$ by way of the local attention network and the global user–item joint representation $P_{global}$ by global attention network, respectively, we need to integrate them together as the input of the subsequent interaction model [43,44]. Here are two kinds of combination methods:

(12) $$P_{total}^{1}=\gamma_{1}\times P_{local}+\gamma_{2}\times P_{global}$$

(13) $$P_{total}^{2}=Concate\left(\gamma_{1}\times P_{local},\gamma_{2}\times P_{global}\right),$$

where $\gamma_{1}\in \left(0,1\right)$ and $\gamma_{2}\in \left(0,1\right)$ are weighted parameters of $P_{local}$ and $P_{global}$. The first method is to add the local user–item joint representation $P_{local}$ and the global user–item joint representations $P_{global}$ weighted by $\gamma_{1}$ and $\gamma_{2}$, and the second method is to use concatenation instead of addition in the first method. Based on these two methods, we design two variants of the model in the experiment section. Both add and concat are common operations used to aggregate feature information in neural networks. The concate operation overlays the dimensions of the feature vector. The information contained in each dimension of the vector does not change, but the dimension of the vector is doubled. The add operation adds the corresponding values of the feature vectors. The dimensions of the vectors do not change, but the information contained in each dimension is increased. Add enriches the representation information for each feature, while concat increases the number of features. After obtaining the combined user–item joint representation $P_{total}$, we need an interaction model to fuse the feature information of representation for generating the rating prediction. The traditional methods mostly use Factorization Machine, which has the advantages of simple operation and low calculation cost. But it can only fuse the first-order and second-order features. So it is difficult to fuse the high-order features. Therefore, in this paper, multi-layer perceptron is adopted as the interaction model, due to its powerful capability of automatically combining high-order features. What is more, the input of multi-layer perceptron model is combined with user–item joint representation $P_{total}$, the output is the prediction rating, defined as follows:

(14) $$y_{pred}=Relu(W^{\left(n\right)}\times \cdots \left(Relu\left(W^{\left(1\right)}\times P_{total}+B^{\left(1\right)}\right)+\cdots B^{\left(n\right)}\right).$$

### 4.4. Model Optimization

The task of this paper is rating prediction based on explicit data. Here, the square loss function is used as the optimization goal [17]:

(15) $$Loss={\left(y_{pred}-y_{real}\right)}^{2}+\epsilon \times {∥Para∥}^{2},$$

where $y_{pred}$ is the prediction rating obtained by the proposed framework, $y_{real}$ is the real rating of user on item, Para are the trainable parameters in the neural network. The first term indicates the difference between $y_{pred}$ and $y_{real}$, and the second term is $L_{2}$ norm regularization, in which the coefficient ϵ is devised to control the regularization intensity to prevent overfitting.

## 5. Experiments

### 5.1. Datasets

To verify the effectiveness of DANER, we performed experiments on two real datasets with rich heterogeneous information. The first dataset was Amazon (https://nijianmo.github.io/amazon/index.html#files accessed on 14 November 2022), which contains reviews (ratings, text, votes), item data (item description, item type, price, brand and image characteristics) and purchase relations from the Amazon e-commerce site. The second dataset is Yelp (https://www.yelp.com/dataset accessed on 14 November 2022), which contains reviews, city information, item attributes, and user characteristics from the American review site Yelp.

The detailed statistics of the datasets used in this paper are shown in Table 2. The Amazon dataset contains 195,791 rating data for 6170 users and 2753 items, and the Yelp dataset contains 19,397 rating data for 16,239 users and 14,284 items. The value of rating ranges from 1 to 5. The higher the value of the rating is, the more attention the user pays to the item. While the lower the rating is, the less interest the user has in the item. All relations used to construct meta-paths have been extracted from the formatted strings in the two original datasets. We use data density to measure data sparseness—the data density calculated as follows:

(16) $$Density=\frac{num(ratings)}{num(users)\times num(items)}.$$

### 5.2. Evaluation Metrics

In order to accurately evaluate the performances of DANER and baseline methods, we adopted two widely used metrics—Mean Absolute Error (MAE) and Rooted Mean Square Error (RMSE)—as the evaluation metrics of our experiment. MAE is the average of absolute error between the prediction value and the real value, which can accurately reflect the actual prediction error. RMSE is the square root of the ratio of the deviation between the prediction value and the real value over the number of samples. RMSE indicates the dispersion of data, which is commonly used as a standard metric for prediction tassk in the machine learning model. MAE and RMSE are defined as follows:

(17) $$MAE=\frac{1}{\left|R_{test}\right|}\sum_{\left(i,j\right)\in R_{test}}\left|\hat{R_{i,j}}-R_{i,j}\right|.$$

(18) $$RMSE=\sqrt{\frac{\sum_{\left(i,j\right)\in R_{test}}{\left(\hat{R_{i,j}}-R_{i,j}\right)}^{2}}{\left|R_{test}\right|}},$$

where $R_{test}$ is a test set of user–item interaction records, $R_{test}$ is the number of user–item interaction records in $R_{test}$, $\hat{R_{i,j}}$ is the prediction rating obtained by DANER, and $R_{i,j}$ is the real rating of user on item. The smaller the values of MAE and RMSE are, the better the recommendation performance is.

### 5.3. Baselines

In order to verify the performance of the proposed framework, the following baseline methods were chosen for comparison.

RegSVD [15]: RegSVD belongs to collaborative filtering method based on users, of which the input is a single user–item rating matrix. It adds $L_{2}$ norm regularization term to constrain the representation vectors of users and items based on matrix decomposition method.

SVD++ [19]: The SVD++ model adds a global average rating, user rating deviation, item rating deviation and user history rating information into the optimization objective function, which achieves a significant improvement on overcoming the problem that the original SVD method does not explicitly consider the impact of a user’s historical behavior on user rating prediction. At present, it has become one of the most typical methods in the field of recommender systems.

NeuCF [45]: NeuCF creatively introduces deep learning into recommendation system and overcomes the shortcomings of inner product operation by using deep neural network as the interaction model between users and items. In addition, NeuCF combines linear and non-linear interaction models to recommend items based on implicit data. In order to apply NeuCF to the rating prediction task of explicit data, the pairwise loss function of the model is replaced by the square error loss function on the basis of the original paper code. Also, the activation function of the neural network is changed to the Relu function.

FMG [30]: FMG introduces heterogeneous information networks as auxiliary information for a recommendation task. In particular, it uses meta-graph to mine the information of heterogeneous networks and adopts matrix decomposition to obtain representations of users and items. Besides, Factorization Machine with group lasso regularization is employed to generate recommendation results. Because the difference of the values in the generated similarity matrices is too large, we add a standardized operation before the matrix decomposition to keep the similarity values in the range of 1 to 5.

### 5.4. Experimental Performance

#### 5.4.1. Experimental Settings

In the experiments, we used Python and TensorFlow deep learning framework to implement the proposed DANER model. In the comparison experiments of model variations and baseline methods, to obtain the optimal experimental performance, the dimension of representation vectors is fixed to 32 for all models. The learning rates and regularization coefficients are tuned in 0.001, 0.005, 0.01, 0.05, 0.1 according to different models. Besides, the drop out ratio is set to 0.5, and the batch size is set to 256.

The parameters of the model were initialized by the uniform distribution initializer. We optimized the model with the Adaptive Moment Estimation optimizer [46]. We also designed an early stopping mechanism to control the training time. When the evaluation metrics or train data loss does not decrease for 20 successive epochs, the training process can be terminated. In order to compare the performances of the models with different training sets, we utilize (80%, 70%, 60%) of Amazon and Yelp datasets for training and the remaining (20%, 30%, 40%) for testing.

#### 5.4.2. Model Variations Comparison Experiments

There are many optional model variations in the modelling process. Therefore, a variety of model variations comparison experiments were designed to find out the best model variation as the final model for the subsequent comparison experiments of baseline methods. Furthermore, through the model variations comparison experiments, we can verify whether the attention mechanism is helpful for improving the performance of recommendation, and can even figure out how the attention mechanism finds the critical meta-path.

In the experiments, five variations of the model with different combination operations on fusing the local and the global user–item joint representations are designed, including the no-attention model (DANER-no), the local-attention model (DANER-local), the global-attention model (DANER-global), the adding-attention model (DANER-add) and the concatenating-attention model (DANER-concate), respectively. The DANER-no does not contain any attention mechanism, and the representations of users and items obtained by matrix decomposition are fed into the interaction model directly. The DANER-local only uses the local attention network after matrix decomposition to obtain the local user–item joint representations as the input of the interaction model. Similar to DANER-local, the DANER-global only uses global attention network between matrix decomposition and interaction model. The DANER-add adopts adding operation when combining the user–item joint representations $P_{local}$ and $P_{global}$. The DANER-concate adopts concatenating operation instead of adding operation in DANER-add. The results of the model variations comparison experiments are shown in Table 3 and Table 4.

According to the experimental results, it is apparent that the performance of DANER-no is the worst over all the variations, which shows that the attention mechanism is helpful to improve the recommendation results. There is no significant gap between the four kinds of models with attention mechanism. However, the performances of DANER-add and DANER-concate are better than those of DANER-local and DANER-global. This may be due to the fact that the latter can mine more feature information of users and items. We can also observe that the DANER-concate outperforms the DANER-add, which shows that the DANER-concate is the best among the five variations. The reason may be that concatenating operation can preserve the feature information of users and items more effectively. Therefore, we use the DANER-concate as the final method of our proposed framework and compare it with the baseline methods. After taking the whole experimental results into account, we can come to the conclusion that the introduction of attention mechanism does improve the accuracy of rating prediction, both of the RMSE and MAE metrics decreasing by more than 5%.

#### 5.4.3. Baseline Methods Comparison Experiments

In this section, we will compare our DANER-concate model with the baseline methods. The results are shown in Table 5 and Table 6.

By observing the experimental results on Amazon and Yelp datasets, it can be found that the larger the proportion of training set is, the smaller the MAE and RMSE metrics of the experiments are, which indicates that the prediction rating is more accurate. This is because the performance of the recommendation task is greatly affected by the sparseness of the rating matrix. As shown in Table 2, the density of the original datasets are very small, and the rating matrices are very sparse. When the proportion of training data increases, the rating matrix of the training set becomes denser. So more rating information can be obtained, which leads to a better performance.

As we can see in Table 5 and Table 6, RegSVD and SVD++ have the worst performance under six experimental conditions of two datasets. They are traditional machine learning methods, which neither use neural networks nor contain auxiliary information of heterogeneous information networks. In addition, SVD++ performs better compared to RegSVD, which may be credited with the fact that SVD++ contains more user history information. In most of the experimental results, NeuCF obviously outperforms the former two methods, except the RMSE metric on the Amazon dataset. It is a deep learning model, which uses neural network as the interaction model to overcome the shortcomings of the previous inner product operation. But because the model is designed for the top-*N* recommendation task of implicit data, it may not be able to play its effect on the rating prediction task of explicit data completely. FMG yields better performance than the previous three methods (RegSVD, SVD++ and NeuCF) which only use the rating information of users and items. Moreover, FMG utilizes meta-graph to extract the information of heterogeneous information networks as auxiliary information, which can help to solve the problem of sparse original rating matrix to a certain extent and get better recommendation performance. In general, the proposed framework DANER surpasses the baseline methods consistently over all conditions in the experiments. The improvement of MAE metric is more remarkable than that of RMSE metric. Specifically, MAE metric of DANER increases by 3.7–4.3% on Amazon dataset and 1.5–2.6% on Yelp dataset. RMSE metric of DANER increases by 0.4–0.8% on Amazon dataset and 1.2–1.7% on Yelp dataset. The reasons for the progress of our framework are as follows: (i). we introduce heterogeneous information networks as auxiliary information to alleviate the data sparseness problem of single rating matrix; (ii). we utilized an attention mechanism to make the generated representations more abundant and effective.

#### 5.4.4. Parameter Comparison Experiments

**Figure 5:**
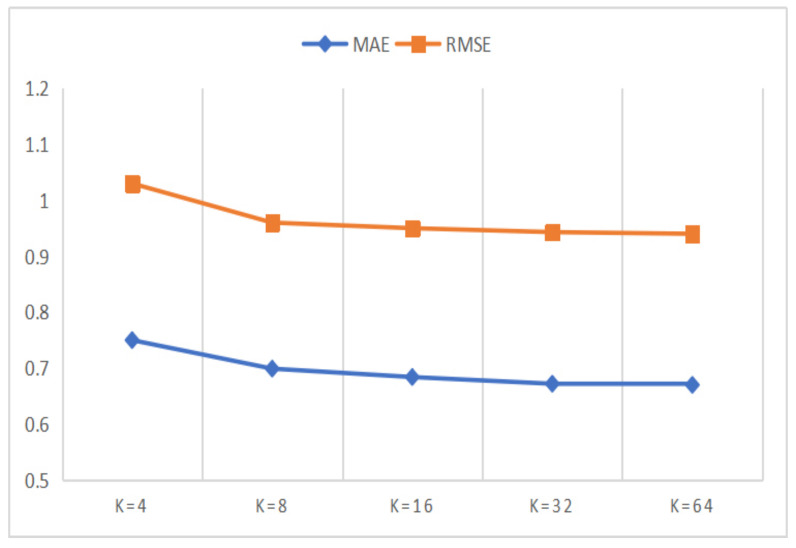
The performances of the DANER model with different *K* on Amazon dataset.

**Figure 6:**
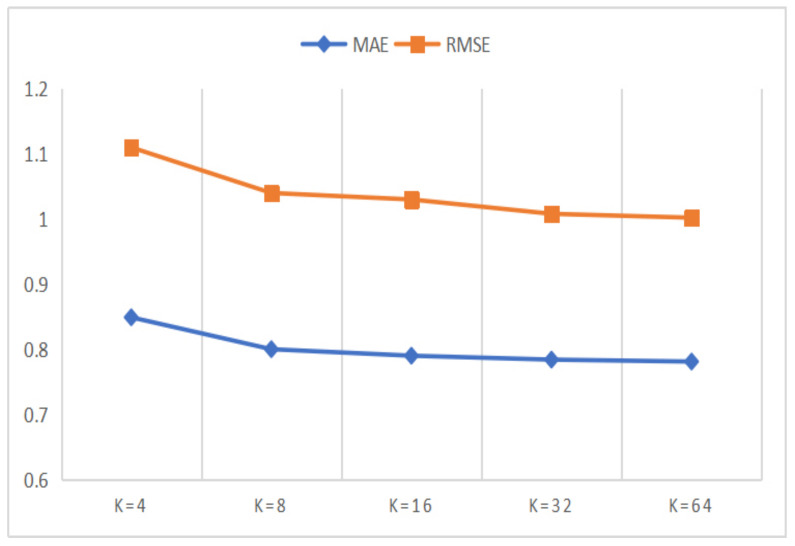
The performances of the DANER model with different *K* on Yelp dataset.

In this section, we designed a series of comparison experiments to study the influence of *K* on the experimental results, where *K* is the dimension of representation vector in matrix decomposition. The value of the dimension *K* affects the amount of information contained in the representation vector. To be specific, the larger the *K* value is, the better the expressive power of the representation vector is. However, larger *K* value will lead to more space consumption and increase the calculation cost of matrix decomposition at the same time. Therefore, in order to get the appropriate *K* value which can balance the performance of the model and the computational cost, we conducted parameter comparison experiments on Amazon and Yelp datasets, where the *K* value waas set to 8, 16, 32 and 64 respectively. The results of the parameter comparison experiments are shown in Figure 5 and Figure 6.

In Figure 5 and Figure 6, we can observe that the experimental performance becomes better with the increase of the *K* value. However, when the *K* value reaches 32, the improvement speed of experimental performance tends to be slow, while the growth speed of the computational cost is more intense. Therefore, considering both the experimental performances and the computational cost of the model, we plan to set the *K* value to 32. At the same time, we can also observe that the MAE and RMSE values for the Amazon dataset are better than for the Yelp dataset. This can be attributed to the fact that the Yelp dataset is more sparse than the Amazon dataset.

#### 5.4.5. Interpretability of Recommendation Results

**Figure 7:**
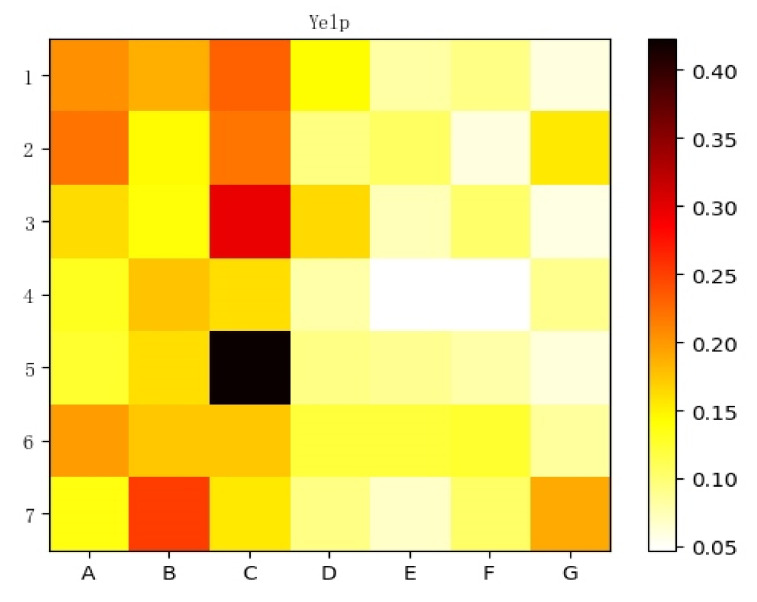
Attention coefficients corresponding to the seven meta-paths of seven records.

**Figure 8:**
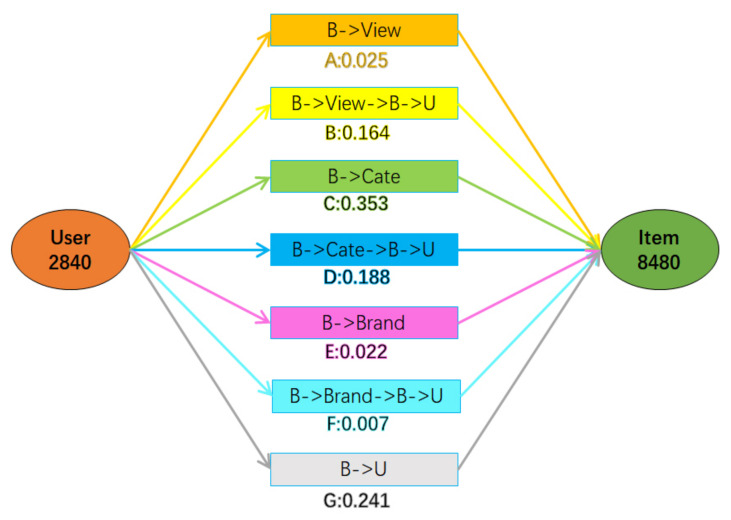
Attention coefficients corresponding to the seven meta-paths in record 5.

The interpretative recommendation results are more reasonable, more persuasive, and more capable of gaining the trust of users. In the process of the experiment, we can obtain the attention coefficients of the global attention network, which provide a basis for further studying the attention mechanism and distinguishing the critical meta-path. Moreover, we can provide an explanation based on the critical meta-path for recommendation results. Specifically, we randomly selected seven groups of user rating records and visualized their global attention coefficients, which are shown in Figure 7 and Figure 8.

The abscissas in Figure 7 are seven predefined meta-paths, which are named *A*, *B*, *C*, *D*, *E*, *F* and *G* respectively. The ordinates are seven pairs of user and item rating records selected randomly. Each block in the figure corresponds to the attention coefficient of the corresponding meta-path in the record, and its numerical value corresponds to the color depth. Figure 8 shows more detailed information about record 5, including the user ID, item ID, meta-path type, and the size of the attention coefficient.

As shown in Figure 8, for the record 5, the user ID is 2840, the item ID is 8480, and the predicted rating of the user for the item is 5, which indicates that user 2840 has a strong desire to buy item 8480. At the same time, the meta-path with the maximum attention coefficient in this record is *C*(U→B→Cate←B). Therefore, we can provide an explanation based on the meta-path U→B→Cate←B, that is, the reason why we recommend item 8480 to user 2840 is that the user 2840 has purchased items with the same category as item 8480.

Based on this situation, several explanations can be specified in advance according to the meta-path. For each result of the recommendation, the explanation corresponding to the meta-path with the largest attention coefficient will be selected as the reason for recommendation.

## 6. Conclusions

In this paper, we proposed a rating prediction framework based on heterogeneous information networks and attention mechanisms. We exploited meta-paths to mine the high-level relationship between users and items in heterogeneous information networks. Then, we adopted a matrix decomposition to generate the latent representations of users and items. After that, we designed local and global attention neural networks to obtain the user–item joint representations integrating multiple meta-path information. By the interaction model, we can obtain the predicted ratings of users on items. The results of several experiments demonstrate that the DANER model is superior to most existing rating prediction models on achieving higher recommendation accuracy. Moreover, we visualized the attention coefficients to explain the recommendation results, which are more trustworthy.

## Figures and Tables

**Table 1:** Meta-paths defined in different datasets.

Dataset	Meta-Path
	U→B
	U→B←U→B
	U→B→Cate←B
Amazon	U→B→Brand←B
	U→B→View←B
	U→B→Cate←B←U→B
	U→B→Brand←B←U→B
	U→B→View←B←U→B
	U→B
	U→U→B
	U→B←U→B
Yelp	U→Com←U→B
	U→B→Cate←B
	U→B→City←B
	U→B→Cate←B←U→B
	U→B→City←B←U→B

**Table 2:** Statistics of Amazon and Yelp datasets.

Amazon		Yelp	
**Entity**	**Number**	**Entity**	**Number**
User	6170	User	16,239
Item	2753	Business	14,284
View	3857	Compliment	11
Category	22	Category	511
Brand	334	City	47
Relation	Number	Relation	Number
user–item	195,791	User-Business	198,397
Item-View	5694	User-User	158,590
Item-Categoty	5508	User-Compliment	76,875
Item-Brand	2753	Business-City	14,267
		Business-Category	40,009
Density = 1.15%		Density = 0.086%	

**Table 3:** Experimental results MAE of model variations comparison experiments. The best results are highlighted in boldface.

Dataset/Ratio	DANER-No	DANER-Local	DANER-Global	DANER-Add	DANER-Concate
Amazon/20%	0.819	0.691	0.69	0.687	**0.672**
Amazon/30%	0.823	0.697	0.696	0.693	**0.689**
Amazon/40%	0.849	0.70	0.701	0.698	**0.695**
Yelp/20%	0.837	0.793	0.792	0.799	**0.784**
Yelp/30%	0.858	0.795	0.805	0.85	**0.795**
Yelp/40%	0.86	0.799	0.809	0.809	**0.798**

**Table 4:** Experimental results RMSE of model variations comparison experiments. The best results are highlighted in boldface.

Dataset/Ratio	DANER-No	DANER-Local	DANER-Global	DANER-Add	DANER-Concate
Amazon/20%	1.066	0.941	0.937	0.935	**0.934**
Amazon/30%	1.071	0.947	0.945	0.945	**0.944**
Amazon/40%	1.075	0.949	0.948	0.95	**0.946**
Yelp/20%	1.065	1.017	1.015	1.016	**1.009**
Yelp/30%	1.073	1.024	1.03	1.025	**1.019**
Yelp/40%	1.08	1.029	1.031	1.028	**1.022**

**Table 5:** Experimental results MAE of baseline methods comparison experiments. The best results are highlighted in boldface.

Dataset/Ratio	RegSVD	SVD++	NeuCF	FMG	DANER
Amazon/20%	0.728	0.715	0.702	0.711	**0.672**
Amazon/30%	0.731	0.726	0.722	0.717	**0.689**
Amazon/40%	0.749	0.781	0.739	0.722	**0.695**
Yelp/20%	0.833	0.818	0.808	0.796	**0.784**
Yelp/30%	0.835	0.819	0.814	0.81	**0.795**
Yelp/40%	0.841	0.825	0.828	0.819	**0.798**

**Table 6:** Experimental results RMSE of baseline methods comparison experiments. The best results are highlighted in boldface.

Dataset/Ratio	RegSVD	SVD++	NeuCF	FMG	DANER
Amazon/20%	0.957	0.949	0.954	0.947	**0.934**
Amazon/30%	0.961	0.954	0.957	0.949	**0.944**
Amazon/40%	0.986	0.966	0.963	0.954	**0.946**
Yelp/20%	1.066	1.051	1.027	1.025	**1.008**
Yelp/30%	1.068	1.054	1.032	1.032	**1.019**
Yelp/40%	1.075	1.060	1.037	1.034	**1.022**

## Data Availability

Amazon: https://nijianmo.github.io/amazon/index.html#files accessed on 14 November 2022; Yelp: https://www.yelp.com/dataset accessed on 14 November 2022.
